# Large-scale transcriptomic analysis reveals that pridopidine reverses aberrant gene expression and activates neuroprotective pathways in the YAC128 HD mouse

**DOI:** 10.1186/s13024-018-0259-3

**Published:** 2018-05-21

**Authors:** Rebecca Kusko, Jennifer Dreymann, Jermaine Ross, Yoonjeong Cha, Renan Escalante-Chong, Marta Garcia-Miralles, Liang Juin Tan, Michael E. Burczynski, Ben Zeskind, Daphna Laifenfeld, Mahmoud Pouladi, Michal Geva, Iris Grossman, Michael R. Hayden

**Affiliations:** 1 Immuneering Corporation, Cambridge, MA 02142 USA; 2 Research and Development, Teva Pharmaceutical Industries Ltd, Netanya, Israel; 3 Translational Laboratory in Genetic Medicine, Agency for Science, Technology and Research, Singapore (A*STAR), Singapore, 138648 Singapore; 4 Centre for Molecular Medicine and Therapeutics, Child and Family Research Institute, University of British Columbia, Vancouver, BC V5Z 4H4 Canada; 5 Department of Medicine, Yong Loo Lin School of Medicine, National University of Singapore, Singapore, 117597 Singapore

**Keywords:** Huntington disease, Movement disorders, Neurodegeneration

## Abstract

**Background:**

Huntington Disease (HD) is an incurable autosomal dominant neurodegenerative disorder driven by an expansion repeat giving rise to the mutant huntingtin protein (mHtt), which is known to disrupt a multitude of transcriptional pathways. Pridopidine, a small molecule in development for treatment of HD, has been shown to improve motor symptoms in HD patients. In HD animal models, pridopidine exerts neuroprotective effects and improves behavioral and motor functions. Pridopidine binds primarily to the sigma-1 receptor, (IC50 ~ 100 nM), which mediates its neuroprotective properties, such as rescue of spine density and aberrant calcium signaling in HD neuronal cultures. Pridopidine enhances brain-derived neurotrophic factor (BDNF) secretion, which is blocked by putative sigma-1 receptor antagonist NE-100, and was shown to upregulate transcription of genes in the BDNF, glucocorticoid receptor (GR), and dopamine D1 receptor (D1R) pathways in the rat striatum. The impact of different doses of pridopidine on gene expression and transcript splicing in HD across relevant brain regions was explored, utilizing the YAC128 HD mouse model, which carries the entire human mHtt gene containing 128 CAG repeats.

**Methods:**

RNAseq was analyzed from striatum, cortex, and hippocampus of wild-type and YAC128 mice treated with vehicle, 10 mg/kg or 30 mg/kg pridopidine from the presymptomatic stage (1.5 months of age) until 11.5 months of age in which mice exhibit progressive disease phenotypes.

**Results:**

The most pronounced transcriptional effect of pridopidine at both doses was observed in the striatum with minimal effects in other regions. In addition, for the first time pridopidine was found to have a dose-dependent impact on alternative exon and junction usage, a regulatory mechanism known to be impaired in HD. In the striatum of YAC128 HD mice, pridopidine treatment initiation prior to symptomatic manifestation rescues the impaired expression of the BDNF, GR, D1R and cAMP pathways.

**Conclusions:**

Pridopidine has broad effects on restoring transcriptomic disturbances in the striatum, particularly involving synaptic transmission and activating neuroprotective pathways that are disturbed in HD. Benefits of treatment initiation at early disease stages track with trends observed in the clinic.

**Electronic supplementary material:**

The online version of this article (10.1186/s13024-018-0259-3) contains supplementary material, which is available to authorized users.

## Background

**Fig. 1:**
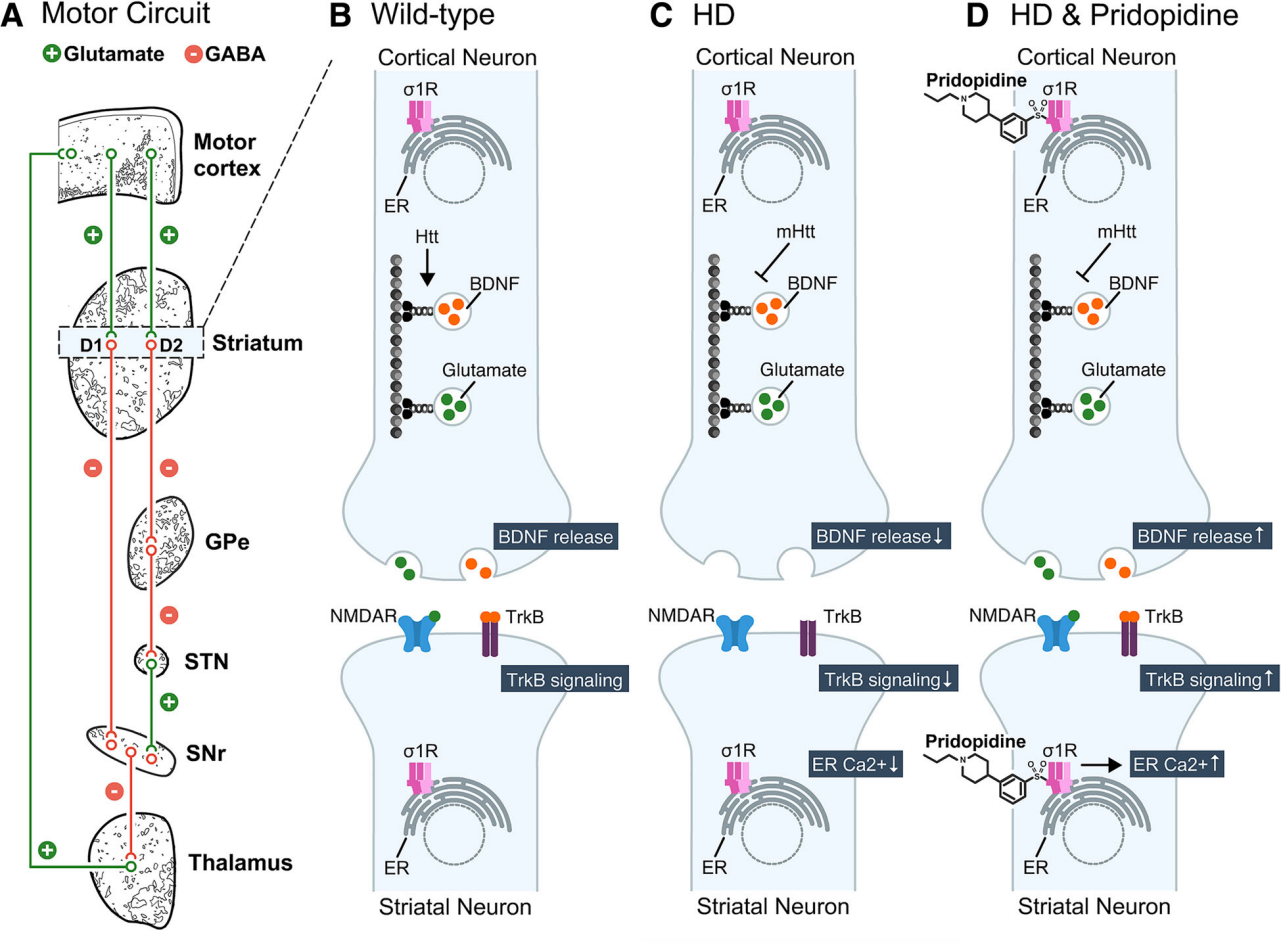
Pridopidine promotes BDNF/TrkB signaling and restores ER calcium levels in the corticostriatal pathway. **a** Shown is a schematic representation of the motor circuit in mammals. Motor cortical neurons project to the striatum and form excitatory (glutamate, green line) synapses with D1 and D2 receptor-expressing neurons (D1 and D2, blue box). Inhibitory D1 receptor-expressing neurons make GABAergic connections (GABA, red line) with the pars reticulata of the substantia nigra (SNr). In contrast, D2 receptor-expressing neurons follow an indirect pathway and send GABAergic projections to the external segment of the globus pallidus (GPe). In turn, GABAergic neurons of the GPe project to the subthalamic nucleus (STN), and excitatory STN neurons send efferents to the SNr GABAergic projections that innervate thalamus, and the thalamus completes the basal ganglia-thalamocortical circuitry by sending excitatory projections to the motor cortex. **b** In the WT striatum, the huntingtin (Htt) protein facilitates axonal transport of synaptic vesicles carrying brain-derived neurotrophic factor (BDNF) and glutamate to the active zone of cortical neurons. Released glutamate and BDNF bind to their targets on the postsynaptic density of striatal neurons, including N-methyl-D-aspartate (NMDA) receptors and tropomyosin receptor kinase B (TrkB) receptors, respectively. **c** In Huntington disease, mutant Htt (mHtt) interferes with the axonal transport process, disrupting normal release of BDNF and consequently TrkB signaling in the striatum. In addition, endoplasmic reticulum (ER) calcium is also perturbed in the striatum during HD progression. **d** Shown is a proposed mechanism of action for pridopidine in the corticostriatal pathway. Treatment with pridopidine has been previously shown to improve both sigma 1 receptor (σ1r)-dependent BDNF release in neuroblastoma cells, increase striatal BDNF levels in HD mice and restore proper ER levels of Ca2+ via direct activation of σ1r in cortical and striatum co-cultures

Huntington Disease (HD) is a progressive and neurological disorder caused by an autosomal dominant CAG trinucleotide expansion in the *Htt* gene [1], characterized by psychiatric, cognitive and motor disturbances, manifesting usually between 40 and 50 years of age and worsening until death [2]. Htt plays a role in facilitating axonal transport of brain-derived neurotrophic factor (BDNF) in the corticostriatal pathway of the motor circuit in wild-type animals (Fig. 1a and b) [3]. Consistently, in animal models of HD, mHtt disrupts several neuronal functions including corticostriatal communication [4] and cortical release of BDNF [5] (Fig. 1c). Breakdown of corticostriatal transmission reduces synaptic activity of striatal neurons [6] and influences downstream signal transduction within the striatum. In addition to the deficiencies in BDNF-TrkB signaling previously reported in mouse models of HD [7, 8], cyclic AMP (cAMP) signaling is disrupted in the striatum of presymptomatic R6/2 HD mice [9].

Pridopidine, a small molecule in development for the treatment of HD, improved motor function in HD patients in two large, double-blind, placebo-controlled studies (HART and MermaiHD) as exhibited by UHDRS–Total Motor Score (TMS), but did not meet primary endpoint of changes from baseline to week 12 in Modified Motor Score [10, 11]. Pridopidine is a high affinity sigma-1 receptor [12] ligand and exerts low-binding affinity towards additional CNS receptors, such as Dopamine D2, Adrenergic a2C, Serotonin 5HT-1A and Histamine H3 [13, 14]. Further, an in-vivo PET imaging study in rats confirmed that pridopidine occupies the sigma-1 receptor at low doses (3 and 15 mg/kg), and the D2R only at higher doses (60 mg/kg). Pridopidine normalizes endoplasmic reticulum (ER) calcium levels in YAC128 corticostriatal co-cultures [15], mediated by the sigma-1 receptor (Fig. 1d). The sigma-1 receptor also mediates pridopidine-induced BDNF in rat neuroblastoma cells [15]. In the striatum of R6/2 HD mice [16, 17], pridopidine treatment increases BDNF protein levels in the striatum (Fig. 1d). Finally, a gene expression analysis in WT rat striatum demonstrates pridopidine induces differential expression (DE) of genes enriched for the BDNF, D1R, and glucocorticoid receptor pathways, presumably mediated via sigma-1 receptor activation.

The effect of different doses of chronic pridopidine treatment, initiated at pre-symptomatic stages, on gene expression and transcript splicing in the context of HD was evaluated using single nucleotide resolution RNA sequencing in YAC128 mice, examining specificity of effects across brain regions.

## Methods

### Animals

YAC128 HD mice [18] (referred to herein as YAC128), maintained on the FVB/N strain were used. Mice were bred and housed according to Garcia-Miralles 2017 [19]. All mouse experiments were performed with the approval of and in accordance with the Institutional Animal Care and Use Committee at the Biomedical Sciences Institute at the Agency for Science, Technology and Research. Pridopidine synthesized by Teva Pharmaceutical Industries was dissolved in sterile water for oral administration. Pridopidine or vehicle was given every day by an oral gavage for 5 days/week for 10 months starting at a presymptomatic stage (1.5 months of age). Mice were split into three treatment groups: vehicle (sterile water), 10 mg/kg of pridopidine (“low dose”), or 30 mg/kg of pridopidine (“high dose”).

A second group of WT mice (C57BI6) were bred and housed at the Department of Experimental Medical Science of Lund University (Sweden), and treated with pridopidine 30 mg/kg for 10 days.

### Sample preparation and RNA extraction

Mice were anaesthetised and perfused with ice-cold phosphate-buffered saline followed by ice-cold 4% paraformaldehyde in phosphate-buffered saline as described in Garcia-Miralles 2017 [19]. Brains were removed from YAC128 and WT, striatum, hippocampus, and cortex were frozen on dry ice, mounted with Tissue-TEK O.C.T. compound (Sakura, Torrance, CA, USA), and sliced coronally into 25-μm sections on a cryostat (Microm HM 525, Thermo Fisher Scientific, Waltham, Massachusetts, USA). The sections were collected and kept in RNAlater solution (Ambion, AM7021) overnight at 4 °C and then stored at − 80 °C until use. Total RNA was isolated by EA Genomics from tissue biopsies from mouse brain regions using the miRNeasy mini kit (Qiagen). RNA was also extracted from blood samples of the same mice using RNeasy Protect Animal Blood Kit EA. RNA integrity was assessed using an Agilent Bioanaylzer and only RNA samples with RIN scores > 8 were used. RNA samples were quantified by NanoDrop for RNAseq.

### RNA sequencing and mapping

EA Genomics performed the RNA sequencing on both mouse studies: 1. Striatum, hippocampus, and cortex of chronic pridopidine or vehicle treated YAC128 or WT mouse and 2. Blood from acute pridopidine treated WT mice. Sequencing was performed using the Illumina TruSeq Stranded mRNA Kit with HiSeq 2x50nt paired end sequencing. Star v.2.5.0a was used to align FASTQ files [20], using the GRCm38 primary assembly annotation and standard options. PCA plots of the samples were used to select outliers and to adjust for possible covariates. Transcripts that had less than 10 reads on average were filtered out. CalcNormFactors from the edgeR R package [21] was used to normalize the counts via the TMM method.

### RNAseq analysis

Following the lead of MAQC [22], the limma v3.28.21 [23] R-package was used to transform and model the gene-level quantification data. Limma::voom was used to transform the count data to log2-counts per million and calculate the mean-variance relationship. Limma::lmFit was used to fit a linear model for each gene based on the experimental design matrix. Limma::eBayes was used to calculate the empirical Bayes moderated t-statistic for contrast significance. Multiple hypothesis adjusted *p*-values were calculated using limma::topTable, which implemented the Benjamini-Hochberg procedure to control FDR. In order to decrease the chance of finding a differential expression signature by chance, we utilized pvalue correction to adjust for the number of hypothesis (genes) we were testing Differential expression contrasts were independently calculated for all three tissues between: **A**. untreated YAC128 and untreated WT samples, **B**. 30 mg/kg pridopidine YAC128 and untreated YAC128 samples, **C**. 10 mg/kg pridopidine YAC128 and untreated YAC128 samples, **D**. 30 mg/kg pridopidine WT and untreated WT samples. In order to compare the magnitude of, and concordance between brain transcriptional signatures and peripheral blood profiles, indicative of the potential to develop biomarkers of disease and response to therapy, we examined samples obtained from a 10-day treatment study, contrasting 30 mg/kg pridopidine WT and untreated WT blood (**E.**).

To test whether the treatment gene expression signature is enriched for relevant pathways, Gene Set Enrichment Analysis (GSEA) [24] was used. All genes tested for differential expression were ranked by limma generated t-statistic for a given contrast. This was input as the “ranked list” in GSEA pre-ranked analysis. Moreover, gene sets were made from lists of differentially expressed genes from literature [25–27] in order to assess whether genes regulated by pridopidine enriched for genes downstream of Dopamine 1 Receptor, BDNF, and Glucocorticoid Receptor. In order to further filter before pathway analysis, we employ a strict version of what is recommended by MAQC, combining a fold change cutoff with an adjusted pvalue cutoff [28]. Hypothesis free broad pathway and transcription factor enrichment was done using Enrichr [29], selecting striatal differential expressed genes combining a fold change with a *p*-value cutoff according to MAQC guidance [28] (absolute linear fold change > 1.25 and adjusted *p*-value < 0.05). For all differential expression, splicing, pathway, and transcription factor analyses we consider “significant” to mean Adj. pval < 0.05 unless otherwise stated.

A signature of genes modulated in HD patient tissue was assembled through a meta-analysis of LIMMA results from two publicly available gene expression datasets from caudate nucleus of 48 total HD patients and 42 controls (GSE26927 and GSE3790). A signature of genes modulated in YAC128 striatum was assembled using LIMMA results from 9 YAC128 mice and 6 WT mice, aged 11.5 months. The HD and YAC128 disease signatures were queried against our expression signatures for pridopidine in striatum of YAC128 mice treated for 10 months at 10 or 30 mg/kg daily using cosine similarity of the moderated t-statistic to assess gene expression reversal.

### Exon and splice junction analysis

Star aligned reads were processed using Quality of RNA-Seq Toolset (QoRTS) with the parameter --stranded. A flat annotation file for GRCm38.p4 was generated using QoRTs and used for subsequent analysis. Differential usage of exon and splice junction (DUEJ) analysis was performed using the JunctionSeq Bioconductor package. JunctionSeq uses a multivariate generalized linear model using a negative binomial distribution to detect exons and splice junctions whose expression changes between conditions relative to the expression of their respective genes. To determine differential usage of exons and splice junctions an adjusted *p*-value cutoff of 0.05 was used.

## Results

### Pridopidine induces striatal gene expression changes in YAC128 HD mice

In a previous study, behavioral and motor effects of pridopidine were evaluated longitudinally, demonstrating improvements in motor coordination, reduced anxiety and depressive like phenotypes, concordant with reversal of specific striatal transcriptional deficits [19]. Here, to characterize the underlying mechanisms, the effect of pridopidine on the YAC128 HD model, gene expression was assessed through the comparison of transcriptomic profiles in YAC128 mice treated with pridopidine (10 or 30 kg/mg, p.o.) or vehicle (5 days/week) and WT mice treated with 30 mg/kg of pridopidine or vehicle (5 days/week). In parallel with the previously described behavioral study [19], animals were treated starting at 1.5 months of postnatal life (presymptomatic) and sacrificed at 11.5 months of age (robust HD phenotype). Gene expression from the striatum, hippocampus, and cortex was evaluated using large RNAseq.

To further identify disease-specific gene expression patterns, vehicle-treated YAC128 mice were compared to vehicle-treated wild-type (WT) mice, demonstrating gene expression changes largely restricted to the striatum. We identified 1346 differentially expressed genes (DEGs) in the striatum (Adj. *p*-val < 0.05, Table 1) compared to 340 DEGs in the hippocampus and 7 DEGs in the cortex (Adj. *p*-val < 0.05, Table 1). Fold change and pvalue ranges are in Additional file 1: Table S1.

**Table 1:** Summary of genes with differential expression or alternative junction/exon usage

Number of Differentially Expressed Genes			
Contrast	Striatum	Hippocampus	Cortex
YAC128 Veh-WT Veh	1346	340	7
YAC128 10 mg/kg-YAC128 Veh	73	0	0
YAC128 30 mg/kg-YAC128 Veh	221	0	0
WT 30 mg/kg-WT Veh	17	0	0
Number of Genes with Alternative Junction/Exon usage			
YAC128 Veh-WT Veh	39	14	4
YAC128 10 mg/kg-YAC128 Veh	1	0	0
YAC128 30 mg/kg-YAC128 Veh	565	1	4
WT 30 mg/kg-WT Veh	0	4	2

An Adj. p-val cutoff of 0.05 was used as pre-requisite criterion to identify differentially expressed genes (top) and alternative exon/junction usage cases (bottom) for each contrast and each tissue

To test if disease progression and/or pridopidine induced gene expression signatures can also be observed outside of the brain, and thus potentially produce biomarkers useful for therapeutic development and monitoring, transcriptomic signals were examined in blood from WT mice treated with pridopidine for 10 days. Only one gene was found to be significantly differentially expressed between 30 mg and vehicle treated mice (IL7R adj pval = 0.03, linear FC 2.11).

In the YAC128 HD model, a dose-dependent effect of pridopidine (vs vehicle) was observed in striatum (Adj. p-val < 0.05, Table 1): 10 mg/kg of pridopidine treatment induced significant differential expression of 73 genes, with 30 mg/kg pridopidine inducing roughly three times as many genes as the 10 mg dose (221 striatal DEGs, Adj. *p*-val < 0.05, Table 1, 55 genes overlap the two lists). No detectible differences in gene expression were observed in the YAC128 hippocampus or cortex after pridopidine treatment (Adj. *p*-val < 0.05, Table 1), suggesting that a robust pridopidine signature is brain compartment specific and not an off target effect. In WT mice, treatment with 30 mg/kg of pridopidine resulted in striatal differential expression of only 17 genes (Adj. *p*-val < 0.05, Table 1). Among these genes, four were also described in a recent microarray study that identified 16 DEGs in the striatum of wild-type rats after treatment with pridopidine (60 mg/kg) [30]. The four overlapping genes are *Junb*, *Egr2*, *Nr4a1*, and *Per1.* With the exception of *Junb,* these genes are also downregulated in the YAC128 mouse model of HD. Taken together, the data demonstrate that the effect of pridopidine on gene expression is more pronounced in a disease model than in WT animals, and is primarily limited to the striatum.

### Pridopidine reverses YAC128 HD mouse model and human HD disease gene expression signatures

**Fig. 2:**
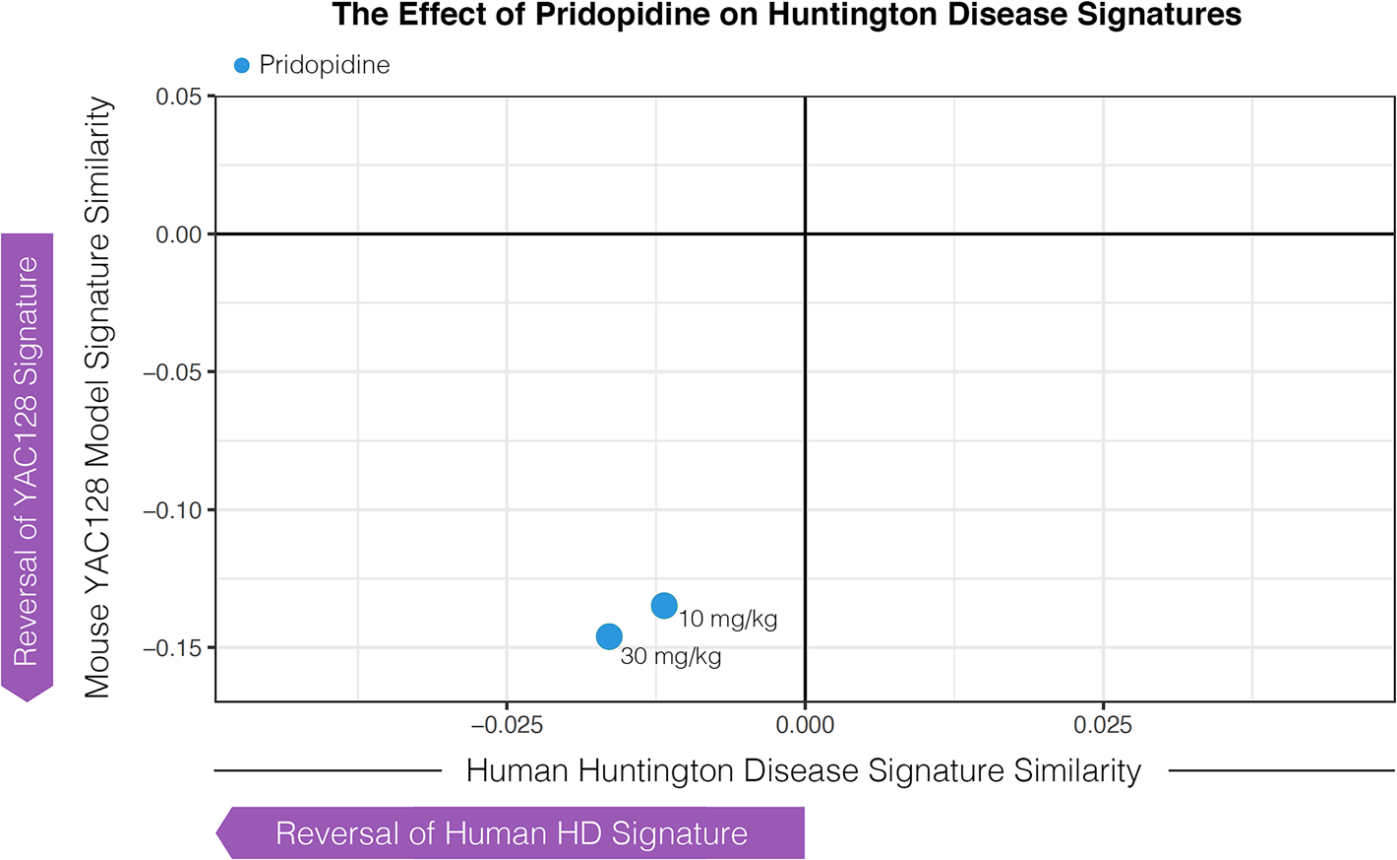
Pridopidine reverses mouse YAC128 and human Huntington disease signatures. Pridopidine reversal of genes modulated in the YAC128 mouse model of Huntington disease (y-axis) as a function of human Huntington disease (x-axis). The blue dots represent 10 and 30 mg/kg dose of pridopidine

We next quantified the extent to which the genes with expression modulated by 10 and 30 mg/kg of pridopidine reverse: 1) genes with expression modulated in HD patient tissue relative to healthy controls (from GSE26927 and GSE3790, described in methods), and 2) genes with expression modulated in YAC128 striatum compared to WT controls. In agreement with Garcia-Miralles 2017 [19], we observed that treatment with 10 and 30 mg/kg of pridopidine significantly reversed the YAC128 disease signature (Fig. 2, Table 2). Moreover, in this study, we additionally observe reversal of our HD patient signature (derived from GSE26927 and GSE3790). The results demonstrate the effectiveness of pridopidine to reverse genes modulated in human HD and the YAC128 mouse model of HD.

**Table 2:** Pridopidine treatment signal significantly reverses YAC128 HD signal. Numbers shown are adjusted *p*-values from Gene Set Enrichment Analysis

Dose	Direction	Striatum	Hippocampus	Cortex
10 mg/kg	Up in YAC128, Down with pridopidine	3.58E-04	3.78E-04	2.40E-04
	Down in YAC128, Up with pridopidine	3.64E-04	4.99E-02	2.40E-04
30 mg/kg	Up in YAC128, Down with pridopidine	3.15E-04	3.46E-04	2.35E-04
	Down in YAC128, Up with pridopidine	3.37E-04	3.53E-03	2.35E-04

Reversal was significant for all doses of pridopidine across the striatum, hippocampus, and cortex of YAC128 mice

**Fig. 3:**
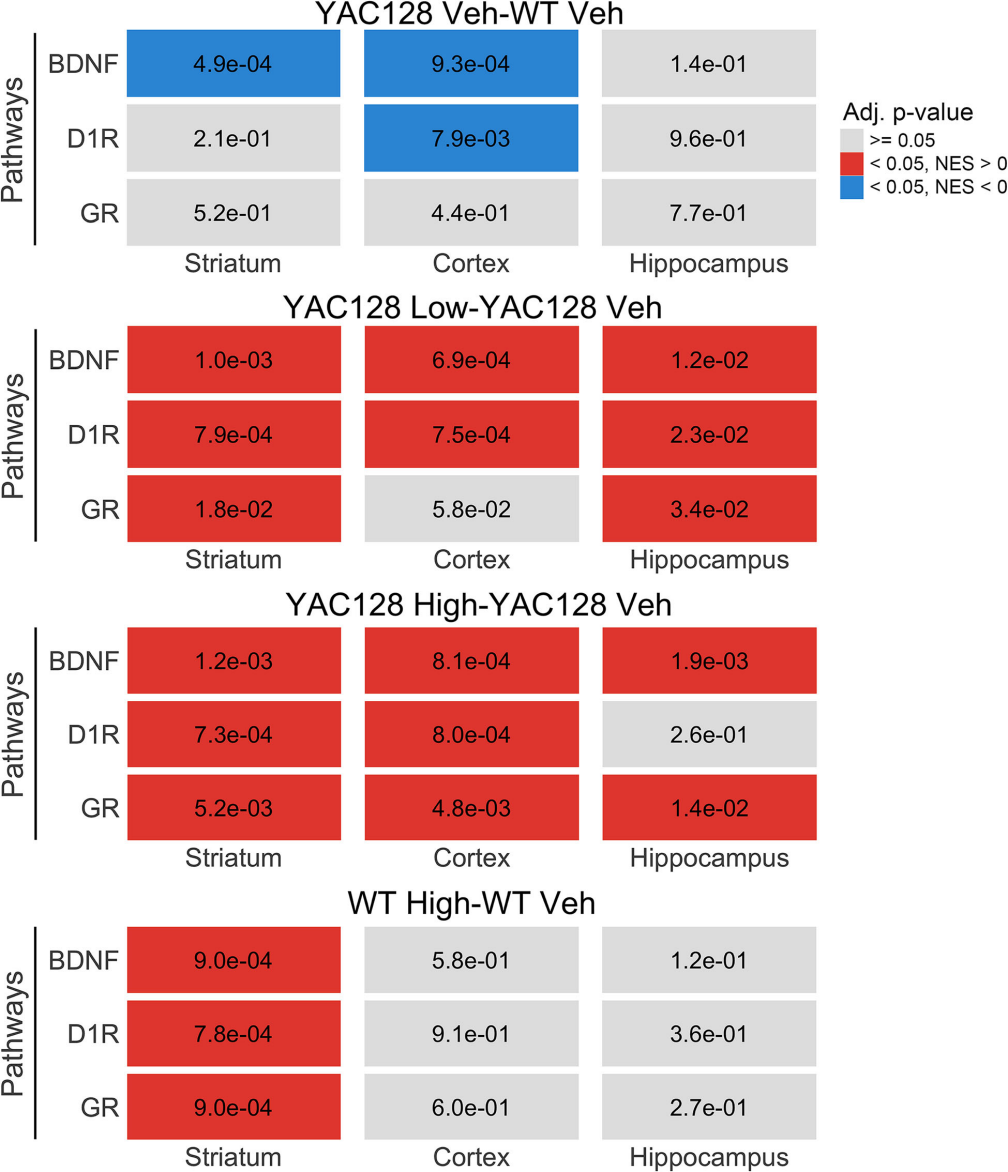
Pathway enrichments for the BDNF, dopamine receptor, glucocorticoid receptor pathways after pridopidine treatment. Each box shows the Adj. p-val for either the BDNF, dopamine D1 receptor (D1R), or glucocorticoid (GR) pathway in a given treatment, genotype, and tissue. Consistent with the gene level results from the RNAseq data, the pathway signal is most consistent in the WT and YAC128 striatum after treatment with pridopidine. “High” = 30 mg/kg of pridopidine; “Low” = 10 mg/kg of pridopidine; “Veh” = vehicle. Grey = no enrichment. Red = significant positive enrichment. Blue = significant negative enrichment

BDNF, GR, and D1R pathways are downregulated in HD [5, 9, 31], while pridopidine upregulates these pathways in WT rat striatum [30]. We investigated whether pridopidine upregulation of these pathways is recapitulated in WT and/or YAC128 mice. We performed Gene Set Enrichment Analysis (GSEA) using manually-curated BDNF, GR, and D1R gene sets. In YAC128 mice, we observed the expected reduction of the BDNF pathway via negative GSEA enrichment in the cortex and striatum, along with downregulation of the D1R pathway in the cortex (Adj. *p*-val < 0.05, Fig. 3). Consistent with previous reports, GSEA pathway analysis revealed positive enrichment of BDNF, GR, and D1R pathway genes in WT mouse striatum after 30 mg/kg pridopidine treatment (Adj. *p*-val < 0.05, Fig. 3). Enrichment analysis also confirmed upregulation of the BDNF pathways in the striatum, hippocampus, and cortex of YAC128 animals after treatment with either the 10 or 30 mg/kg dose of pridopidine (Adj. p-val < 0.05, Fig. 3). In addition, D1R and GR pathways were positively enriched across all three tissues after either 10 or 30 mg/kg treatment of pridopidine, with two exceptions: no significant enrichments were observed 1) for the GR pathway in the cortex after 10 mg/kg treatment; and 2) for the D1R pathway in the hippocampus after 30 mg/kg treatment.

### Database-driven enrichment analysis reveals that pridopidine enhances relevant biological pathways altered in the YAC128 HD striatum

**Fig. 4:**
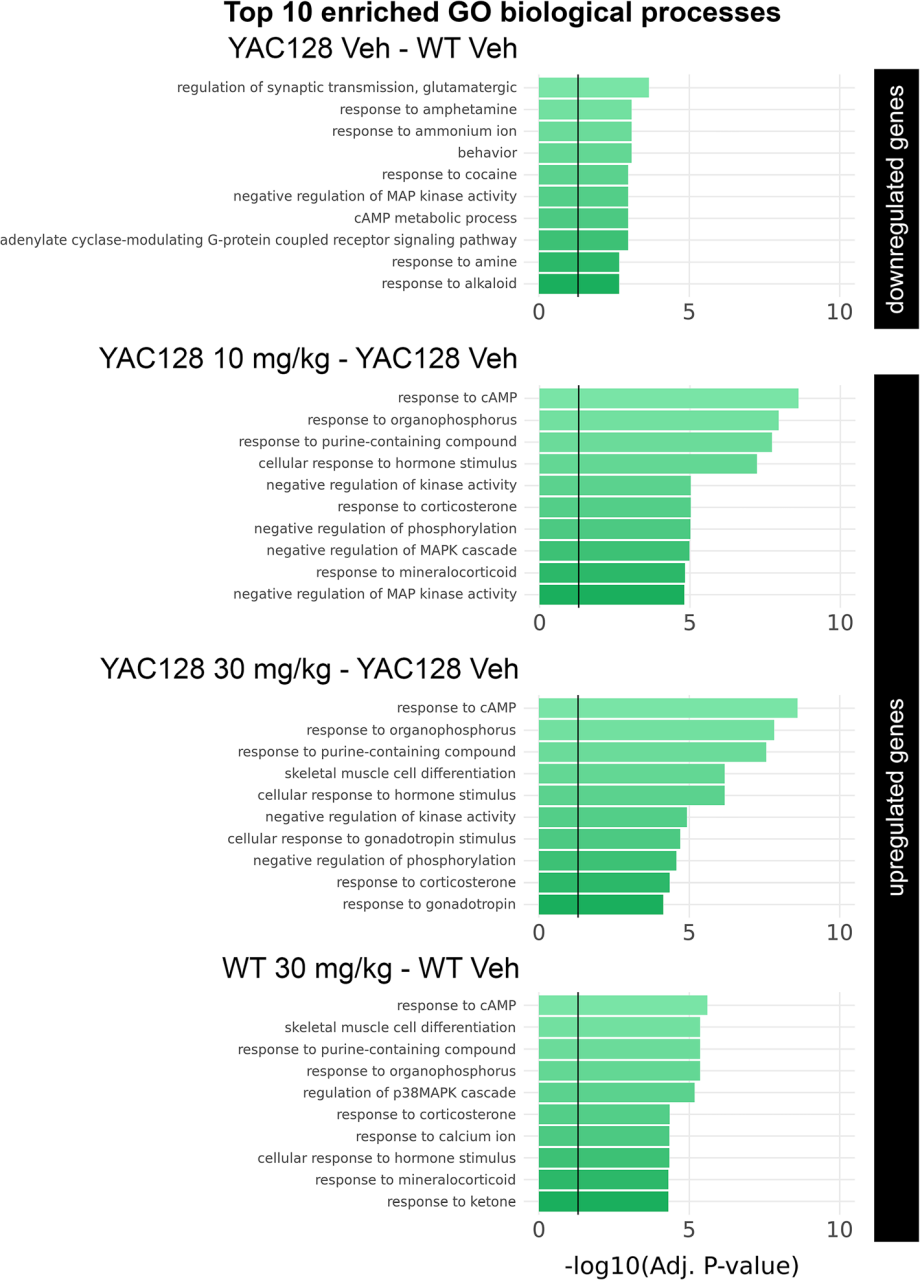
Top 10 enriched Gene Ontology (GO) biological process pathways. Pathway enrichment was performed on striatal DEGs downregulated in vehicle-treated YAC128 mice (top panel), striatal DEGs upregulated after treatment with 10 mg/kg pridopidine in YAC128 (second panel), striatal DEGs upregulated after treatment with 30 mg/kg pridopidine in YAC128 (third panel), and striatal DEGs upregulated after treatment with 10 mg/kg pridopidine in WT (second panel)

While GSEA provides a robust approach for pathway analysis, the method is limited to the manual curation of gene sets. To expand systematically our search for biological processes modulated by pridopidine, we next employed the enrichment tool Enrichr, which utilizes several comprehensive datasets including the Gene Ontology (GO) database. Our analysis focused on DEGs identified in the striatum, where the effect of pridopidine was most pronounced. Enrichment analysis was performed to identify pathways that are downregulated in vehicle-treated YAC128 and upregulated in pridopidine-treated YAC128 mice. Top 10 (of 63) downregulated pathways in the YAC128-vehicle mice (Adj. p-val < 0.05, Additional file 2: Table S2), include impaired synaptic transmission processes, MAP kinase activity, cAMP metabolism, and adenylate cyclase signaling, as well as response to amphetamine and cocaine (Adj. *p*-val < 0.05, Fig. 4).

Pridopidine treatment upregulated cAMP and kinase activity pathways impaired in the YAC128 striatum. Enrichment analysis revealed upregulation of enhanced biological pathways in the YAC128 striatum, with the cAMP response biological process showing a robust, statistically significant signal, ranking 1st after treatment with either the 10 or 30 mg/kg dose of pridopidine (Adj. p-val = 2.49E-09 and 2.53E-09 respectively, Fig. 4). For both doses of pridopidine, other highly ranked biological processes include negative regulation of kinase and phosphorylation (Adj. p-val < 0.05, Fig. 4 and Additional file 2: Table S2). In the YAC128 striatum, there were no significant GO enrichments using DEGs 1) upregulated after vehicle treatment, or 2) downregulated after pridopidine treatment. In wild-type mice, pridopidine induced enrichment of cAMP and p38-MAPK regulation pathways (Adj. *p*-val < 0.05, Fig. 4, Additional file 2: Table S2).

To identify enriched transcriptional factors (TFs) constituting upstream regulatory mechanisms, ENCODE and ChIP Enrichment Analysis (ChEA) databases were queried via Enrichr using DEGs that were: 1) downregulated in the YAC128 versus WT striatum, or 2) upregulated in the pridopidine treatment group. TF analysis of genes downregulated in YAC128 striatum revealed enrichment for gene targets of the transcription factor SUZ12 (Adj. p-val < 0.05, Additional file 3: Table S3). These enrichments are consistent with previous studies showing that SUZ12 is perturbed due to epigenetic dysregulation in HD [32].

TF analysis of DEGs upregulated in the pridopidine treatment group revealed CREB1 transcription factor as a highly significantly enriched gene set (10 and 30 mg/kg) in the YAC128 striatum (Adj. p-val < 0.05, Additional file 3: Table S3). This is consistent with both disrupted CREB activity in HD [33] and the identification of upregulation of cAMP-response genes after pridopidine treatment (Adj. p-val < 0.05, see Additional file 2: Table S2). CREB1 gene set was also enriched after 30 mg/kg pridopidine treatment in WT striatum (Adj. *p*-val < 0.05, Additional file 3: Table S3). Taken together, these observations suggest that pridopidine treatment may induce gene expression regulated via the CREB transcriptional pathway, known to be disrupted in HD.

### Pridopidine reverses compromised cAMP response gene activity in the YAC128 HD striatum

**Fig. 5:**
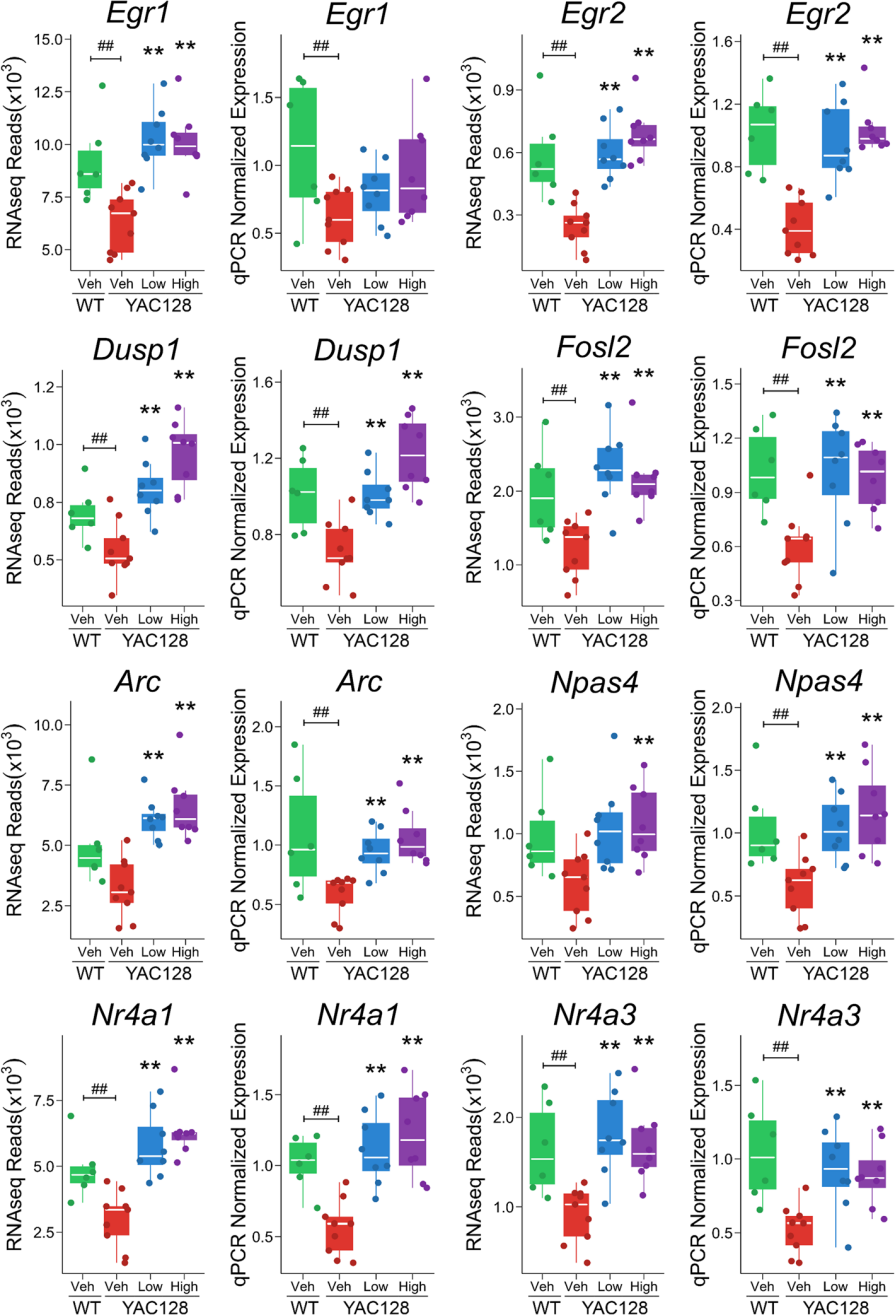
qPCR validation of differential expression of striatal genes in YAC128 mice. Shown are RNAseq and qPCR results for genes differentially expressed in the striatum of YAC128 mice after pridopidine treatment. “**” and “##” represent significant (Adj. p-val < 0.05) differential expression in YAC128 Veh-WT Veh and YAC128 Low/High-YAC128 Veh contrasts, respectively. “High” = 30 mg/kg of pridopidine; “Low” = 10 mg/kg of pridopidine; “Veh” = vehicle

**Fig. 6:**
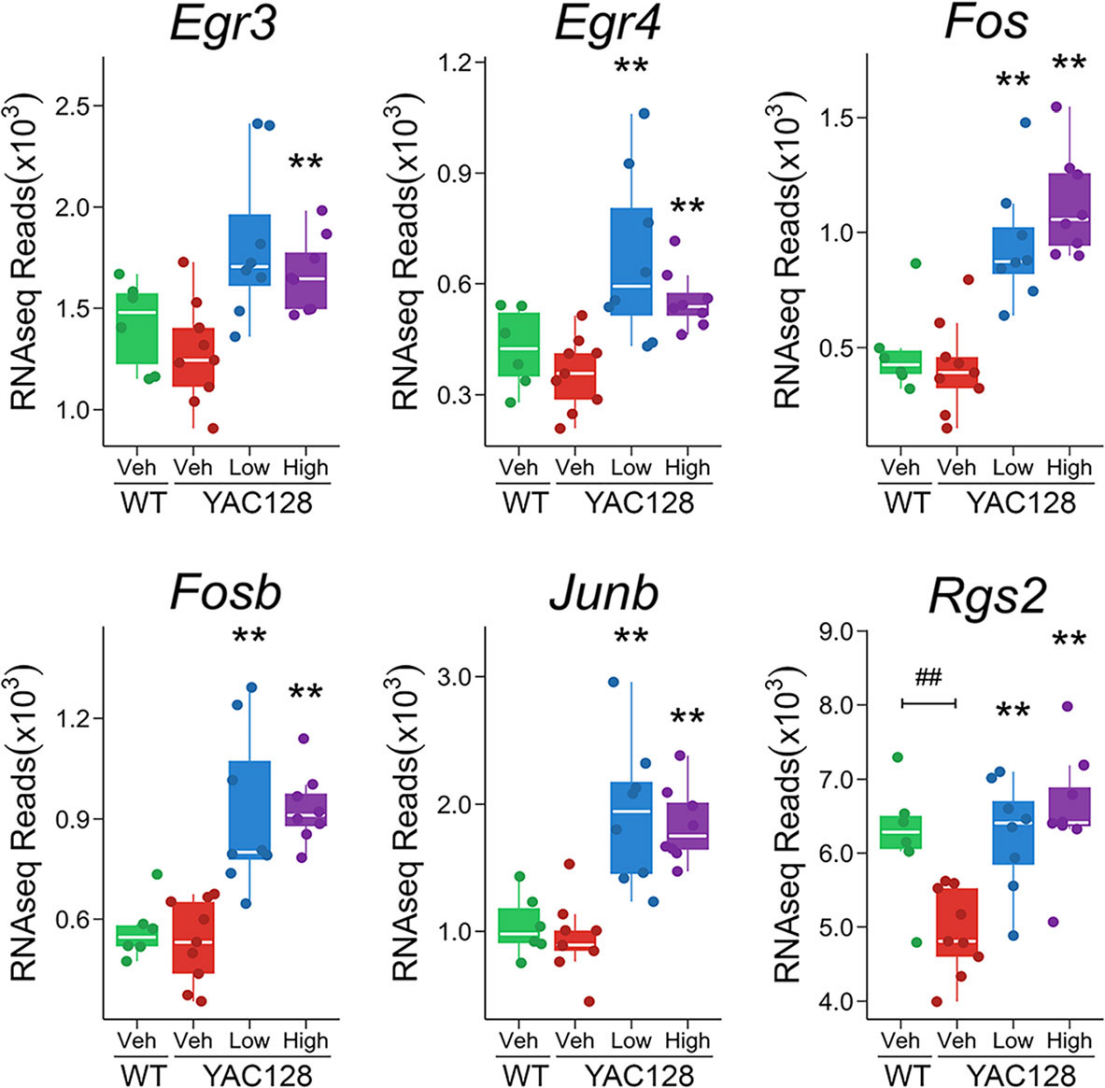
RNAseq differential expression analysis of cAMP-related genes in the YAC128 striatum. Shown are RNAseq results for differentially expressed genes in the YAC128 striatum after pridopidine treatment. “**” and “##” represent significant (Adj. p-val < 0.05) differential expression in YAC128 Veh-WT Veh and YAC128 Low/High-YAC128 Veh contrasts, respectively. “High” = 30 mg/kg of pridopidine; “Low” = 10 mg/kg of pridopidine; “Veh” = vehicle

In YAC128 striatum, pridopidine enrichment of cAMP response is composed of 8 upregulated genes (*Dusp1*, *Egr1*, *Egr2*, *Egr4*, *Fos*, *Fosb*, *Fosl2*, and *Junb*) after treatment with 10 mg/kg of pridopidine, and 9 upregulated genes (*Dusp1*, *Egr1*, *Egr2*, *Egr3*, *Egr4*, *Fos*, *Fosb*, *Fosl2*, and *Junb*) after treatment with 30 mg/kg of pridopidine (Adj. p-val < 0.05, Figs. 5 and 6). In addition, we also identified another cAMP-regulated gene, *Rgs2* [34], upregulated after pridopidine treatment (Fig. 6). Five of these genes (*Dusp1*, *Egr1*, *Egr2*, *Fosl2, and Rgs2*) are downregulated in the striatum of vehicle-treated YAC128 mice (Adj. p-val < 0.05, Fig. 5, Additional file 4: Table S4). qPCR confirmed pridopidine reversed the expression of *Dusp1*, and *Egr2*, and *Fosl2* (Adj. p-val < 0.05, Fig. 5 and Additional file 4: Table S4).

### Pridopidine modulates exon and transcript junction in the striatum of HD mice

Alternative splicing represents a key transcriptomic regulatory mechanism required for many basic cellular functions. Recent evidence suggests alternative splicing may be perturbed in HD. To determine if alternative splicing also occurs in YAC128 mice, we performed differential usage of exon and splice junction (DUEJ) analysis in the striatum, hippocampus, and cortex. In YAC128 mice compared to WT controls, we observed 39, 14, and 4 DUEJs in striatal, hippocampal, and cortical genes, respectively (all Adj. p-val < 0.05, Table 1). In concordance with gene level differential expression, the majority of DUEJ occurred in the striatum.

We then examined whether treatment with pridopidine compared to vehicle induces DUEJ in the brains of WT and YAC128 mice. In the YAC128 striatum, pridopidine induced dose-dependent DUEJ, with 565 genes significant in the 30 mg/kg group compared to only a single gene in 10 mg/kg pridopidine-group (both Adj. p-val < 0.05, Table 1). In WT mice, treatment with pridopidine did not lead to any DUEJ differences in striatum (all Adj. p-val < 0.05, Table 1). Eleven genes (*Kifap3*, *Zwint*, *Cltc*, *Rtn1*, *Acin1*, *Ano3*, *Dclk1*, *Ppp3ca*, *Atp2b2*, *Arpp19*, and *Arpp21*) demonstrated significant DUEJ and reversal after 30 mg/kg pridopidine treatment in the YAC128 striatum (Adj. *p*-val < 0.05). Pridopidine induced minimal to no DUEJ changes in hippocampus and cortex (Table 1). These results are consistent with the dose-dependent effect of pridopidine on gene expression restricted to the YAC128 striatum.

**Fig. 7:**
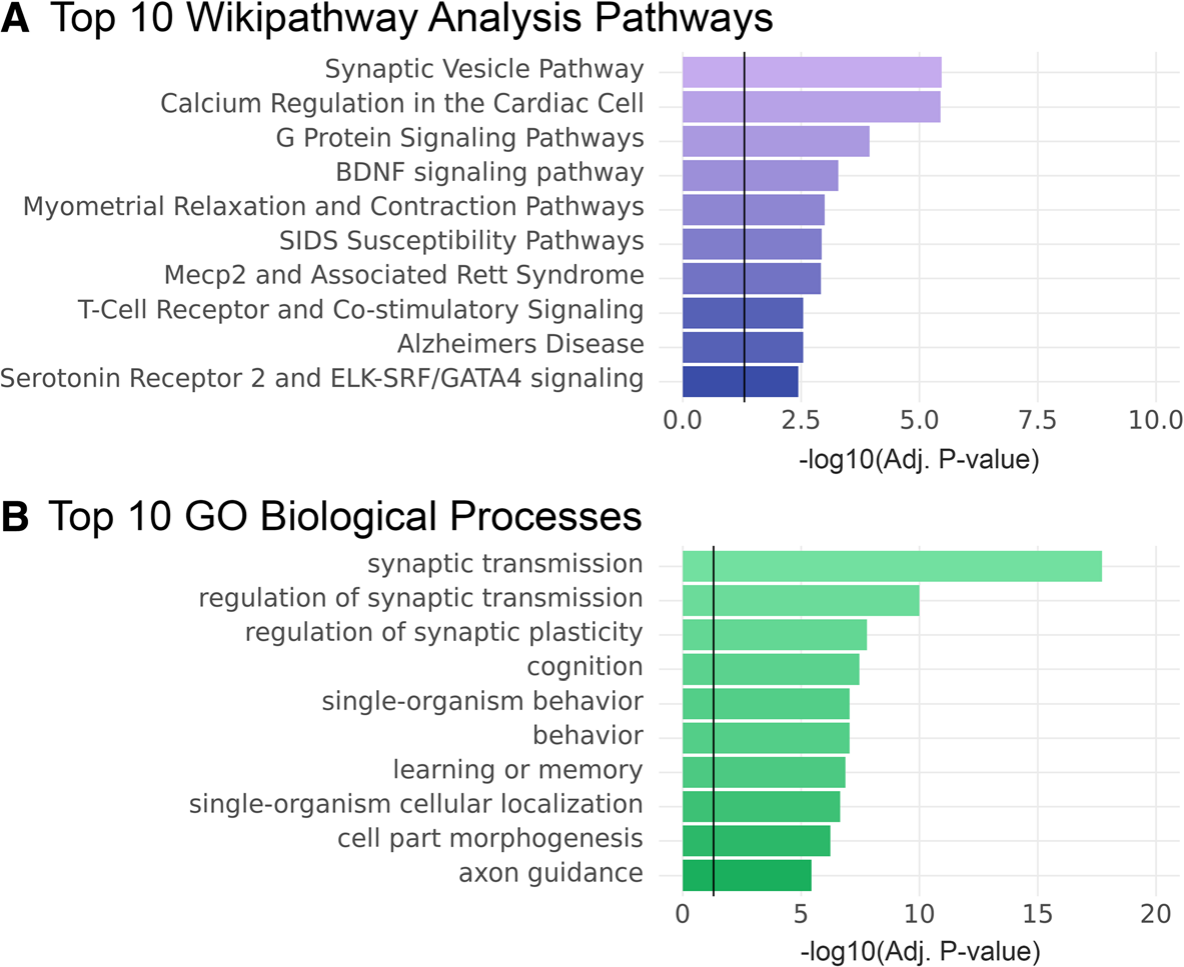
Pathway and differential exon/splice junction analysis in the YAC128 striatum after pridopidine treatment. Pathway analysis was performed on genes that demonstrated pridopidine induced alternative exon/junction usage at a high dose (30 mg/kg). **a** Top 10 significant (Adj. *p*-val < 0.05) pathways from the WikiPathway database. **b** Top 10 significant (Adj. *p*-val < 0.05) pathways from the Gene Ontology (GO) pathway database

Pathway analysis on the 565 genes demonstrating DUEJ after 30 mg/kg pridopidine treatment in YAC128 mice showed enrichments for pathways previously described to be involved in pridopidine’s mechanism of action such as calcium regulation (adj.pval = 3.6E-06), and Synaptic Vesicle (3.4E-06). Additional pathways of interest previously reported as part of pridopidine’s mechanism of action include: BDNF signaling and G protein signaling, (Adj. *p*-val < 0.05, Fig. 7a, b and Additional file 5: Table S5). Taken together, these results demonstrate that the 30 mg/kg of pridopidine induces exon and junction level changes in pathways that are relevant to HD pathology.

## Discussion

It has recently been demonstrated that 30 mg/kg of pridopidine rescues motor behavioral deficits in YAC128 mouse model of HD [19]. To identify potential mechanisms by which pridopidine confers motor benefits, this study focuses on pridopidine induced changes in transcription across multiple brain regions and dose regimens. To characterize the functional relevance of transcriptomic changes, RNAseq data was analyzed for expression signaling, as well as splice variant modifications in pre-specified pathways, as well as across the genome unbiasedly. Testing was performed on WT and YAC128 striatum, cortex, and hippocampus after treatment with vehicle, 10 or 30 mg/kg pridopidine from a presymptomatic stage through disease progression. Both doses of pridopidine had a significant effect on gene and transcript levels in the striatum, with modest to unobserved effects in the cortex and hippocampus. However, the transcriptional effect of pridopidine in YAC128 striatum is dose-dependent. The two doses tested herein suggest linearity of the effect, which future studies employing additional doses will serve to shed further light on. While this study cannot directly query whether pridopidine’s behavioral benefits are transcriptionally mediated, the fact that pridopidine’s main transcriptomic effect is detected in the striatum supports this hypothesis. Moreover, the dose dependent functional expression signals induced by pridopidine track well with the dose-dependent behavioral benefits induced it induces at parallel experimental conditions (Garcia-Miralles et al., 2017). Lastly, genes with perturbed expression in YAC128 pathology are oppositely modulated by pridopidine in the striatum, far more so than expected by chance.

The transcriptional footprint of pridopidine demonstrates a reversal of the disease-specific gene expression and alternative splicing. The disease mechanisms reversed by pridopidine include critical neuroprotective pathways such as BDNF, D1R and glucocorticoid pathways previously reported. qPCR confirmed differential expression of many genes in these pathways. In addition, pridopidine induced gene expression triggered by cAMP transduction, also supported by modulation of downstream transcription factors (e.g. CREB1). Together, these findings provide robust data to demonstrate pridopidine restores mechanisms impaired in HD, specifically in the striatum.

Previous studies reported rescue of several aspects of HD, including phenotype and behavior, in the YAC128 mouse through BDNF overexpression [35]. Dexamethasone, a glucocorticoid that activates the GR pathway, also dampens disease progression in a HD animal model [36]. Both 10 and 30 mg/kg pridopidine treatment in YAC128 mice significantly induced the BDNF, GR and D1R pathways in the striatum, hippocampus, and cortex, consistent with prior reports in WT rat [30]. As pridopidine does not directly bind GR (internal data, not shown), it suggests that the upregulation of the GR pathway may be indirect. Pridopidine increases dopamine efflux in the striatum [37], which may explain the observed upregulation of expression for D1R pathway genes after pridopidine treatment.

**Fig. 8:**
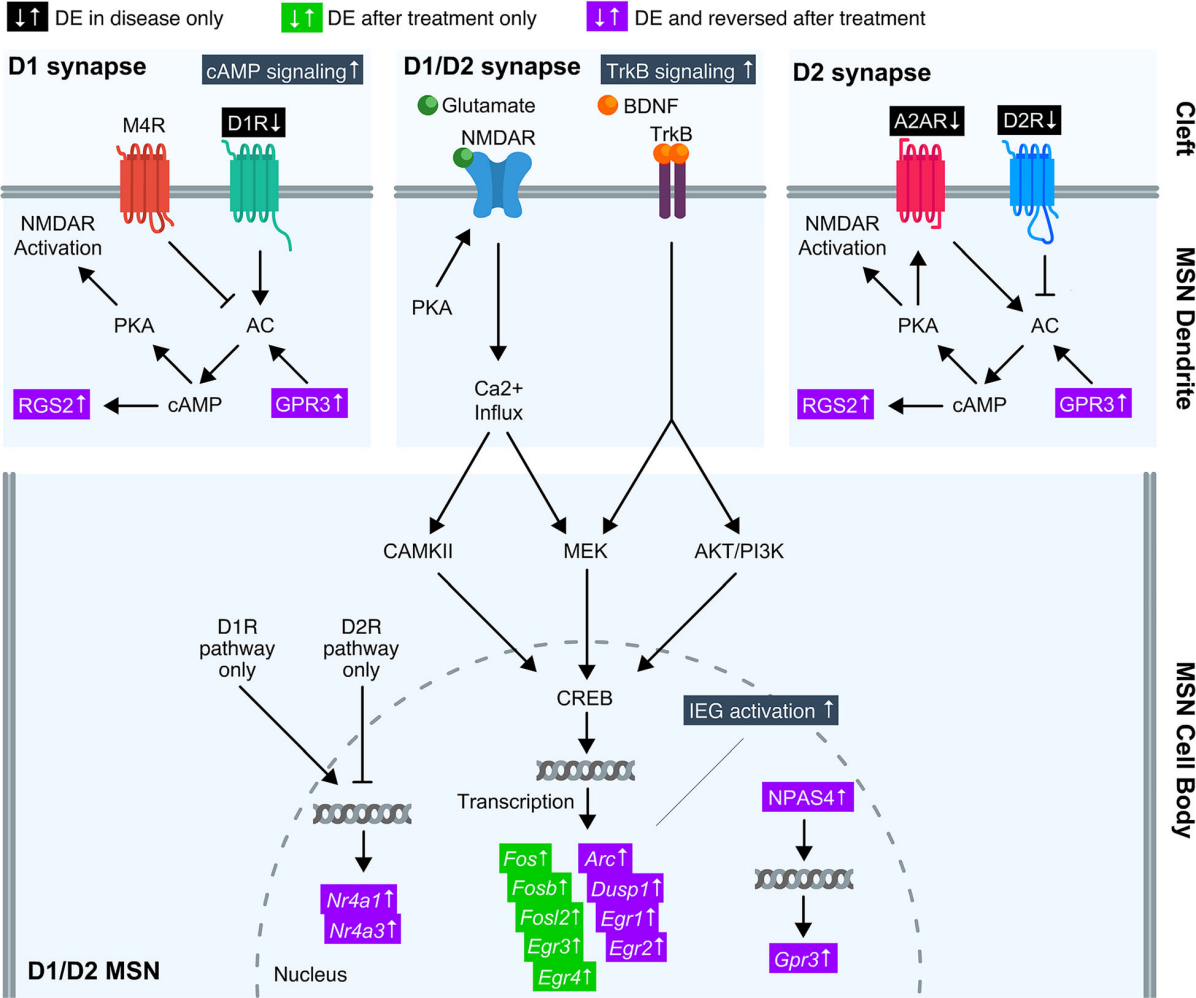
Pridopidine enhances cAMP/PKA and TrkB pathway genes in the YAC128 striatum. Shown is a model of gene regulation after treatment with pridopidine in the YAC128 striatum. Dopamine transmission directs the activation of dopamine D1 and D2 receptors (D1R and D2R, respectively) in medium spiny neurons (MSNs) of the striatum. On the WT postsynaptic density of a D1 synapse, D1Rs activate adenylyl cyclase (AC) in MSNs, whereas muscarinic acetylcholine receptor M4 (M4R) inhibits AC activity. In contrast, D2Rs negatively regulate AC in MSNs, while A2ARs are AC agonists. GPR3 activates AC in both D1R and D2R-expressing MSNs, where RGS2 is a target of cAMP signaling. Activation of AC is upstream of cAMP and PKA, which augments NMDAR activity. Dopamine D1 and D2 receptor genes (*Drd1* and *Drd2*) and A2AR gene (*Adora2a*) are differentially expressed (DE) and downregulated in the YAC128 striatum, but unchanged after pridopidine treatment (black highlighting). Both *Rgs2* and *Gpr3* are downregulated in YAC128 striatum (Adj. p-val < 0.05) and upregulated after treatment of pridopidine. NMDARs and TrkB receptors are expressed in both D1R and D2R-expression MSNs. NMDAR and TrkB receptors both indirectly activate the transcription factor (TF) CREB in the nucleus via downstream pathways. In turn, CREB activates gene expression of several targets. Genes *Arc, Dusp1, Egr1* and *Egr2* are downregulated in the YAC128 striatum, but upregulated after pridopidine treatment (Adj. p-val < 0.05). *Fos*, *Fosb*, *Fosl2*, *Egr3*, and *Egr4* are unperturbed in the striatum of YAC128 mice, but expression of these genes is restored after treatment with pridopidine (Adj. *p*-val < 0.05). Dopamine pathway genes *Nr4a1* and *Nr4a3* and TF gene *Npas4* are downregulated in YAC128 and upregulated after pridopidine treatment (Adj. *p*-val < 0.05). NPAS4 regulates *Gpr3* gene expression

In the WT striatum, dopamine is a central regulator of cAMP activity in both D1 and D2 receptor-expressing neurons, namely, medium spiny neurons, where D1Rs and D2Rs have opposing effects on cAMP levels [38]. Previous studies of HD postmortem brain tissue and animal models have shown that cAMP signaling becomes deregulated in the striatum of humans and animal HD models [9, 39, 40]. Restoration of cAMP levels reduced mHtt aggregates in the striatum of R6/2 HD mice [40], underscoring the importance of rescuing striatal cAMP signaling. In the YAC128 striatum, we observed downregulation of cAMP pathway genes, which are upregulated after treatment with pridopidine (Figs. 5, 6, and 8).

In agreement with Garcia-Miralles et al. [19], we noted reversal of compromised expression of dopamine receptor genes (*Drd1* and *Drd2*) after pridopidine treatment in the YAC128 striatum. However, reversal of *Drd1* and *Drd2* expression was only nominally significant after treatment with either the 10 or 30 mg/kg dose of pridopidine [19]. Therefore, pridopidine could partly restore dopamine-cAMP signaling via compensatory mechanisms. One possibility is that pridopidine induces post-translational regulation of the D1R protein. PSD-95 has been shown to increase D1R surface level expression [41], regulate D1R internalization, and D1R-cAMP signaling [42, 43]. In the striatum, wild-type HTT binds to postsynaptic density protein 95 and promotes its clustering (PSD-95) [42, 44], whereas mHTT lacks binding affinity to PSD-95 [44]. In agreement with increased D1R activity, compromised expression of D1R-regulated genes *Nr4a1* and *Nr4a3* is rescued after treatment with either 10 or 30 mg/kg pridopidine in the YAC128 striatum (Adj. *p*-val < 0.05, Fig. 5).

In addition to dopamine signaling, other signal transduction pathways regulate cAMP response targets, which may also explain the putative effect of pridopidine on cAMP signaling. For example, we identified two additional cAMP-related DEGs (*Npas4* and *Gpr3*) upregulated after treatment with pridopidine in the YAC128 striatum (Additional file 6: Figure S1). Activity-dependent NPAS4 has been shown to upregulate both *BDNF* and *Gpr3* expression in cultured excitatory and inhibitory neurons, respectively [45]. GPR3 is a constitutive activator of cAMP signaling via adenylyl cyclase (AC) [46, 47]. Interestingly, *Npas4* gene expression is compromised in the YAC128 striatum, but rescued after pridopidine treatment with either dose (10 or 30 mg/kg, Adj. *p*-val < 0.05, Fig. 5). In addition, *Gpr3* gene expression is downregulated in the YAC128 striatum, whereas striatal expression of *Gpr3* is upregulated after either 10 or 30 mg/kg pridopidine treatment in YAC128 mice (Additional file 5: Figure S5, Adj. p-val < 0.05). Taken together, this suggests that pridopidine could partly rescue dysregulated cAMP signaling by modulating *Npas4* and *Gpr3* gene expression. In addition to the GPR3-cAMP pathway, BDNF-TrkB signaling has also been shown to activate cAMP response element binding (CREB) protein activity and thus facilitate gene expression in cultured striatal neurons [26]. In other words, pridopidine may induce transcription by binding to S1R, which leads to enhanced BDNF activity, in turn activating gene and splice-variant expression. This alternative mechanism is also supported by the fact that pridopidine induces BDNF release in neuroblastoma cells [30]*.*

Recently, it was demonstrated that treatment with 30 mg/kg of pridopidine rescues motor deficiencies in YAC128 mice, whereas no effect was detected in YAC128 animals treated with 10 mg/kg of pridopidine [19]. In agreement with this observation, we report a broader effect of 30 mg/kg pridopidine on gene expression in the YAC128 striatum compared to the 10 mg/kg dose, but also report that either dose reverses disease associated gene expression. The effect of 30 mg/kg pridopidine on motor function diminishes during the progression of the disease [19], and the lowest locomotor performance is observed between 10 and 12 months of age when mice are very ill. Given that RNA samples for this study were collected during the decline of motor activity in 30 mg/kg pridopidine-treated YAC128 mice, it is difficult to correlate improvement in motor deficit and pridopidine-induced gene expression in the striatum of YAC128 animals. Moreover, our study showed that pridopidine induces a robust gene expression signal when treatment begins early in disease course. This may suggest that in humans, pridopidine may be more effective if started in early disease stages. For both of these reasons, a longitudinal study with earlier time points would better illuminate the link between gene expression and motor behavior after treatment with pridopidine in YAC128 mice.

## Conclusions

In conclusion, pridopidine reverses HD associated changes in transcription at the pathway, gene and splice-variant level. Pathways with transcriptomic aberrations in the YAC128 mouse that are restored to WT levels by pridopidine treatment include BDNF, D1R, GR, cAMP, and calcium signaling. These pathways together are known to interact, and likely positively feed into each other downstream of pridopidine treatment, to relieve HD associated motor symptoms. Beneficial effects when treatment is initiated early, before symptoms are manifest, tracks with trends observed in clinical trials. Studying the effect of pridopidine at multiple time points over the course of treatment against transcriptomic aberrations in YAC128 will reveal additional regulatory dynamics. The results in this study, all taken together, support exploring pridopidine’s role as a therapeutic for neuroprotection in HD and similar neurological movement disorders.

## Additional files

Additional file 1: **Table S1.** Adjusted p-val range and fold change range for differential expression and DUEJ genes meeting adj p-val < 0.05 cutoff. (XLSX 11 kb)

Additional file 2: **Table S2.** Gene Ontology pathway analysis of genes differentially expressed in the mouse striatum. (XLSX 27 kb)

Additional file 3: **Table S3.** Transcription factor enrichment analysis of genes differentially expressed in the mouse striatum. (XLSX 18 kb)

Additional file 4: **Table S4.** qPCR validation of striatal gene expression identified in RNAseq and pathway analysis. (XLSX 10 kb)

Additional file 5: **Table S5.** Pathway analysis of alternatively spliced genes identified after high dose treatment with pridopidine. (XLSX 31 kb)

Additional file 6: **Figure S1.** Pridopidine reverses downregulation of *G Protein-Coupled Receptor 3* (*Gpr3*) gene expression in the striatum of YAC128 mice. Shown are RNAseq results for Gpr3 in the YAC128 striatum after pridopidine treatment. “**” and “##” represent significant (Adj. p-val < 0.05) differential expression in YAC128 Veh-WT Veh and YAC128 Low/High-YAC128 Veh contrasts, respectively. “High” = 30 mg/kg of pridopidine; “Low” = 10 mg/kg of pridopidine; “Veh” = vehicle. (PDF 151 kb)

## Abbreviations

AC: Adenylyl cyclase
BDNF: Brain-derived neurotrophic factor
cAMP: Cyclic AMP
ChEA: ChIP Enrichment Analysis
CREB: cAMP response element binding
D1R: Dopamine D1 receptor
DE: Differential expression
DUEJ: Differential usage of exon and splice junction
ER: Endoplasmic reticulum
GO: Gene ontology
GPe: External segment of the globus pallidus
Gpr3: G protein-coupled receptor 3
GR: Glucocorticoid receptor
GSEA: Gene set enrichment analysis
HD: Huntington disease
Htt: Huntingtin
M4R: Muscarinic acetylcholine receptor M4
MAQC: MicroArray quality control
mHtt: Mutant Htt
MSNs: Medium spiny neurons
NMDA: N-methyl-D-aspartate
QoRTs: Quality of RNA-Seq toolset
SNr: Pars reticulata of the substantia nigra
STN: Subthalamic nucleus
TF: Transcriptional factors
TMS: Total Motor Score
TrkB: Tropomyosin receptor kinase B
WT: Wild-type
σ1r: Sigma-1 receptor
