# Supplementary figures and images for: Large-scale transcriptomic analysis reveals that pridopidine reverses aberrant gene expression and activates neuroprotective pathways in the YAC128 HD mouse
Source: Mol Neurodegener. 2018 May 21;13:25. doi: 10.1186/s13024-018-0259-3 (PMC5963017; doi:10.1186/s13024-018-0259-3)

# *Gpr3*

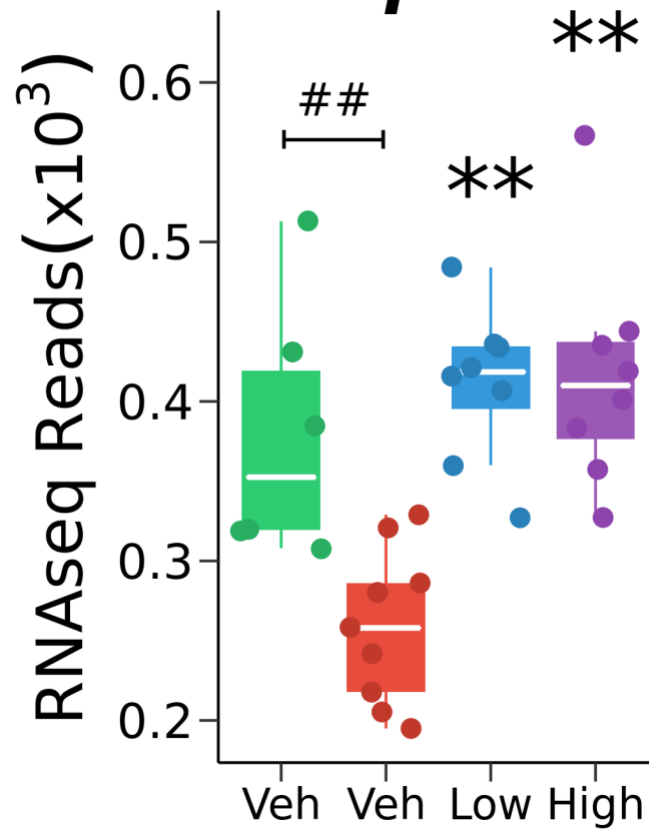

Supplement: Supplementary file 6 — Figure S1. Pridopidine reverses downregulation of G Protein-Coupled Receptor 3 (Gpr3) gene expression in the striatum of YAC128 mice. Shown are RNAseq results for Gpr3 in the YAC128 striatum after pridopidine treatment. “**” and “##” represent significant (Adj. p-val < 0.05) differential expression in YAC128 Veh-WT Veh and YAC128 Low/High-YAC128 Veh contrasts, respectively. “High” = 30 mg/kg of pridopidine; “Low” = 10 mg/kg of pridopidine; “Veh” = vehicle. (PDF 151 kb) [file 13024_2018_259_MOESM6_ESM.pdf]
